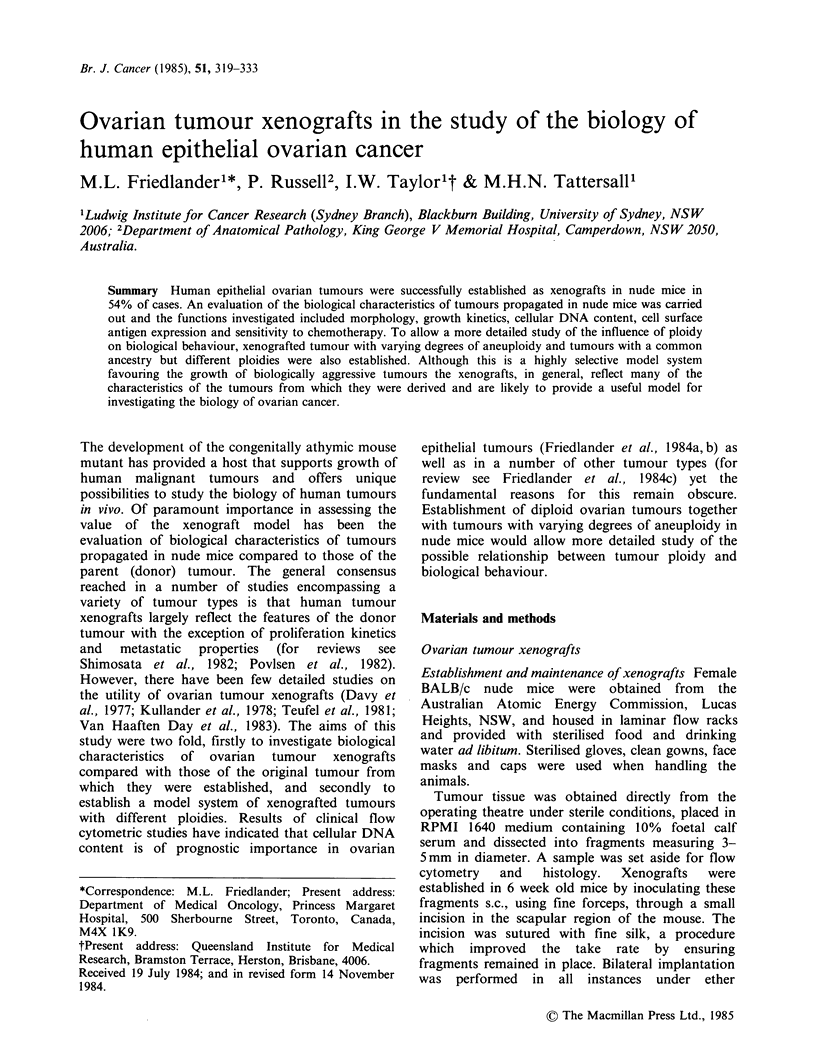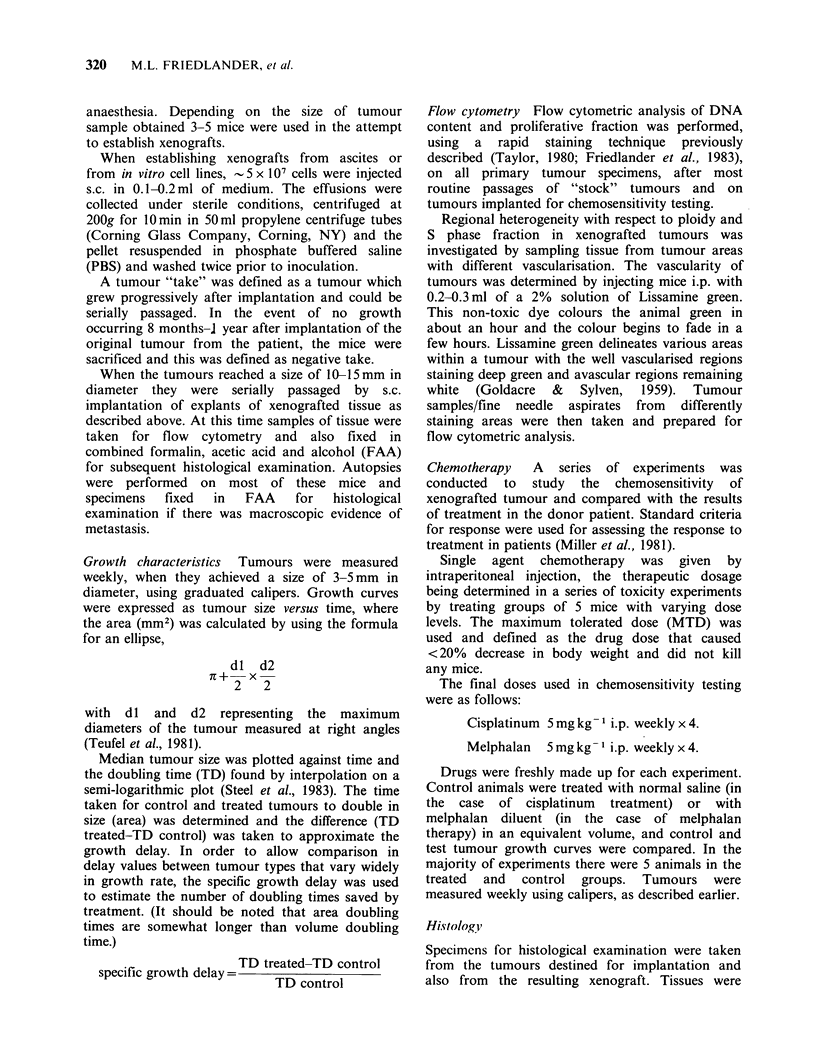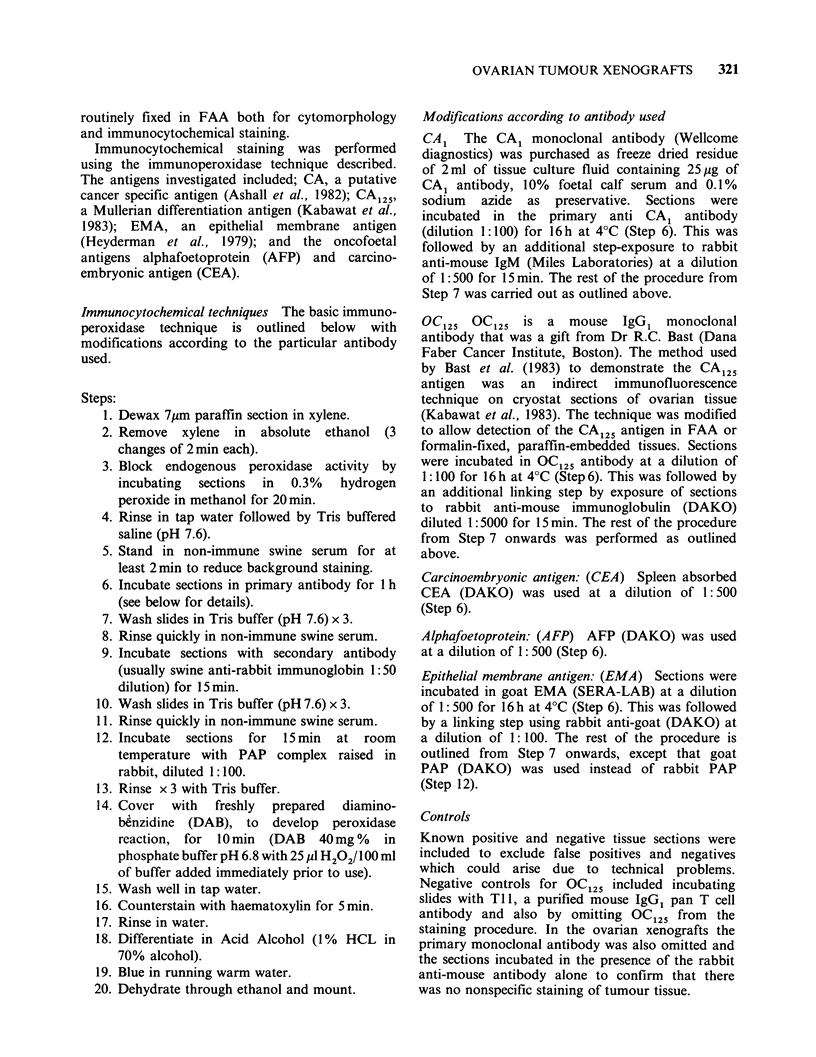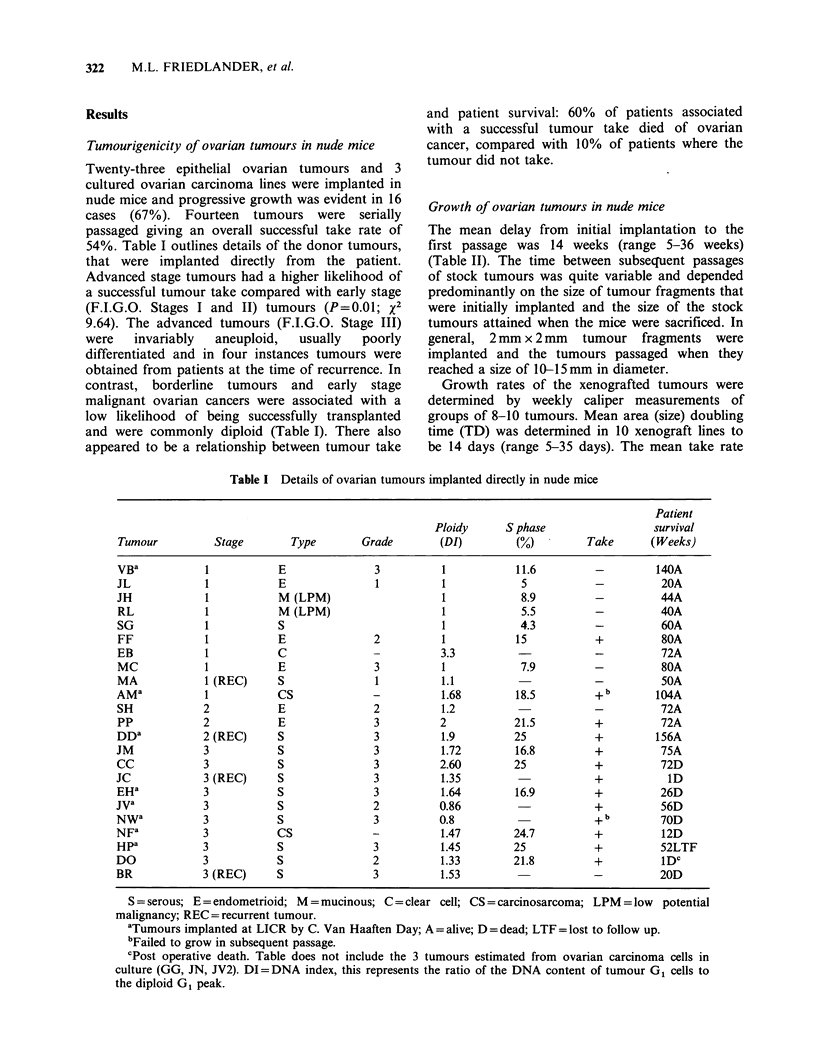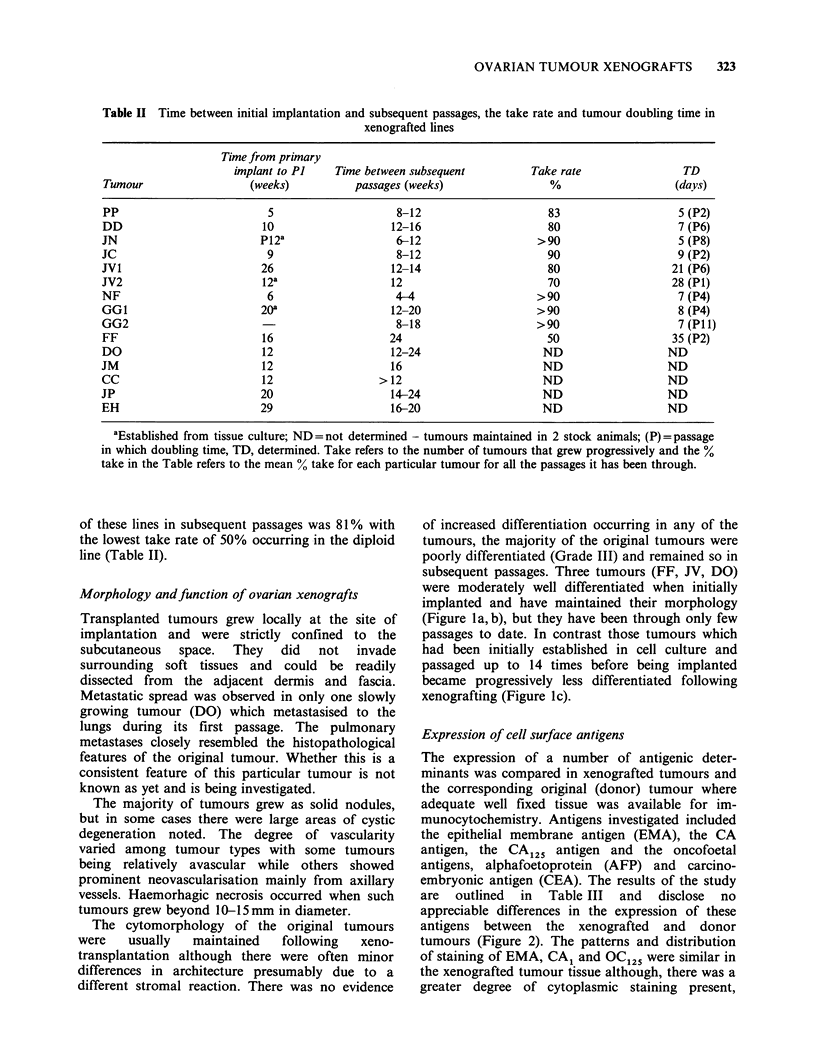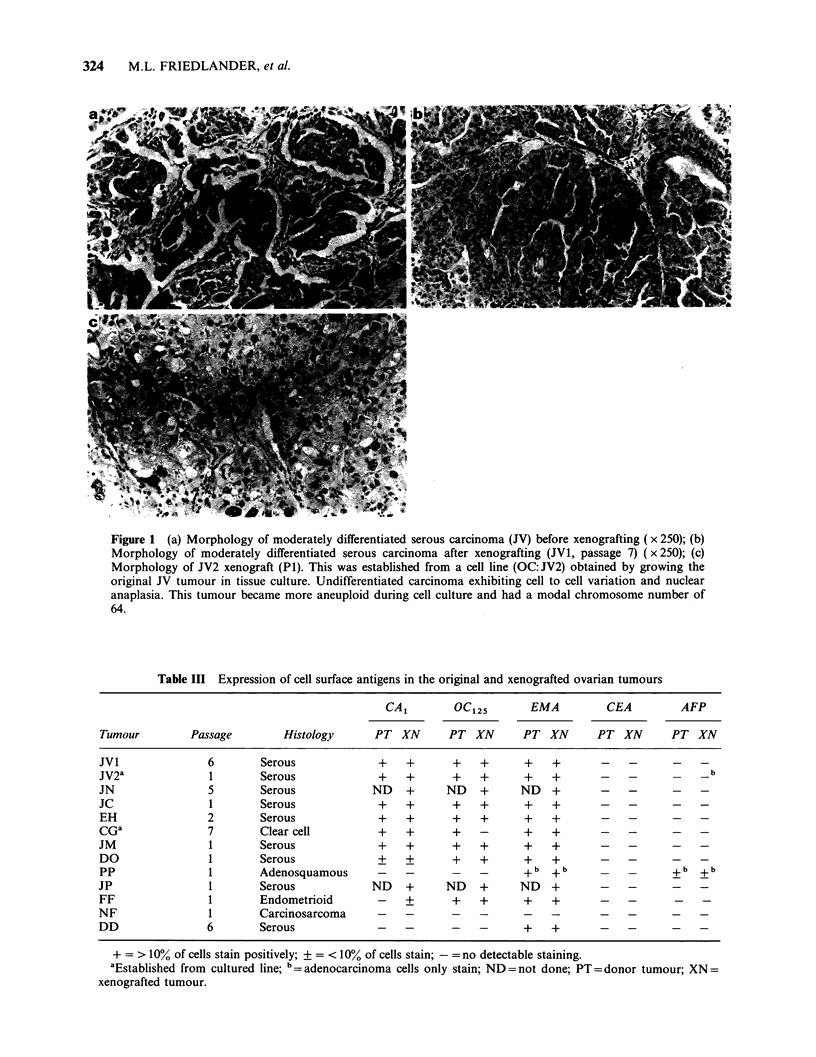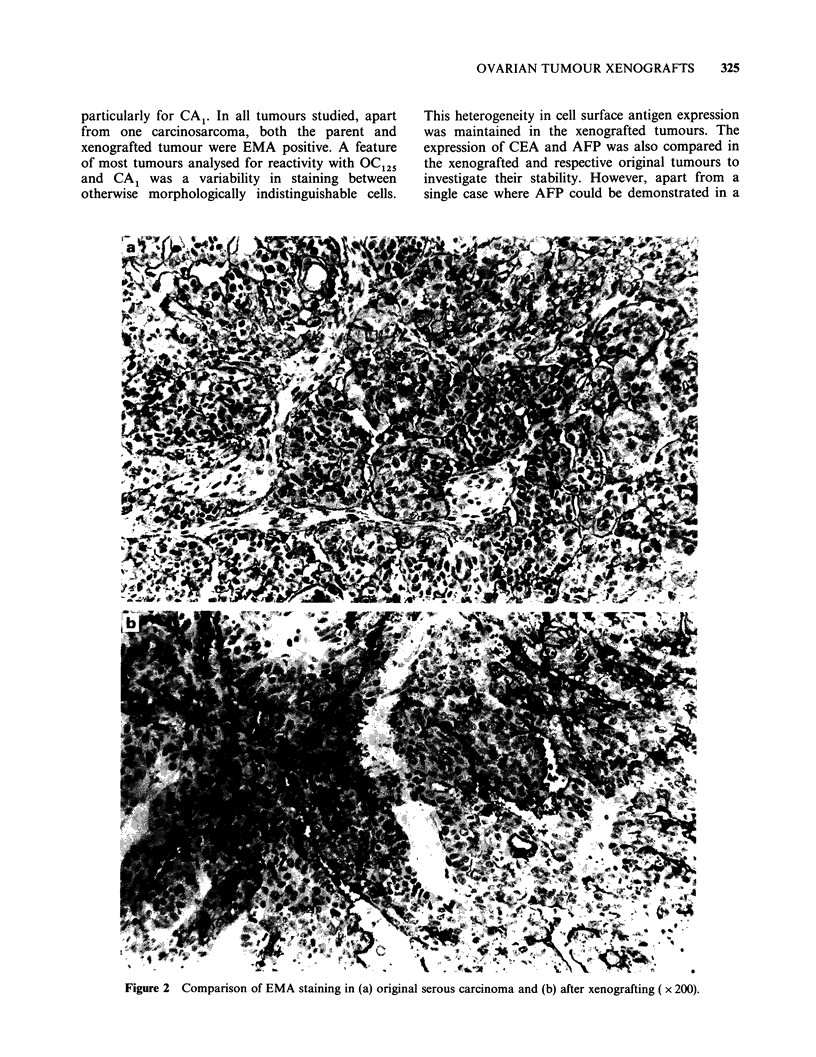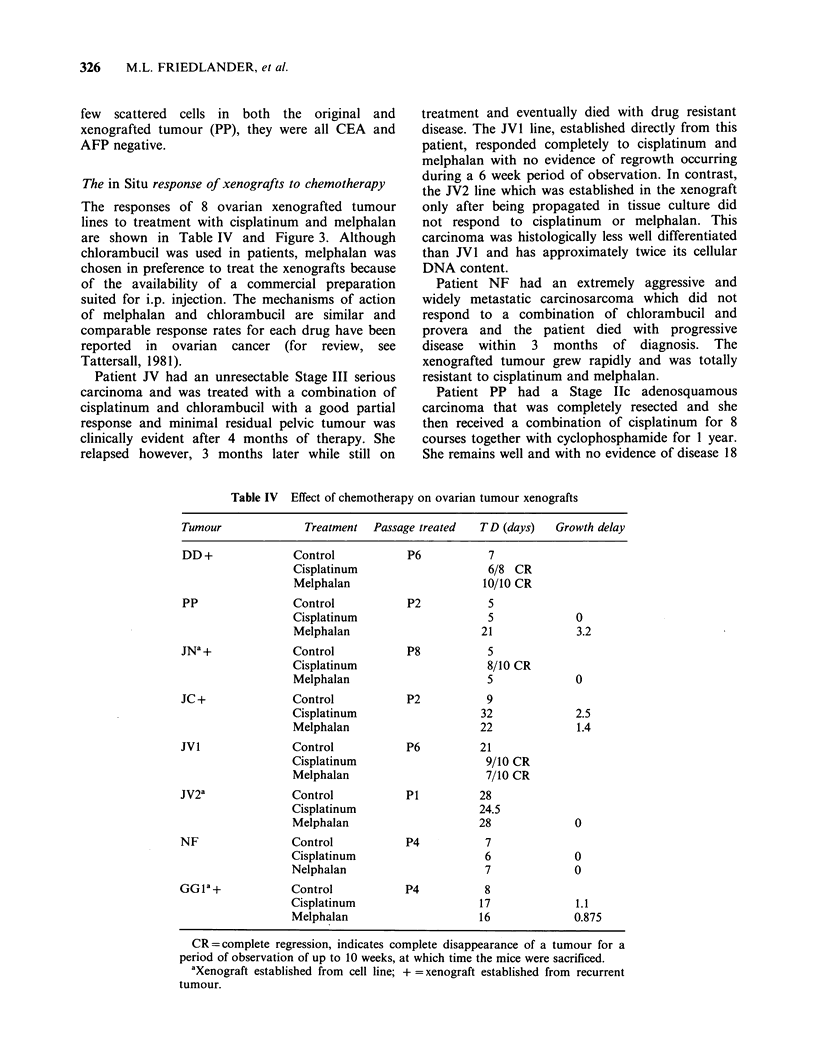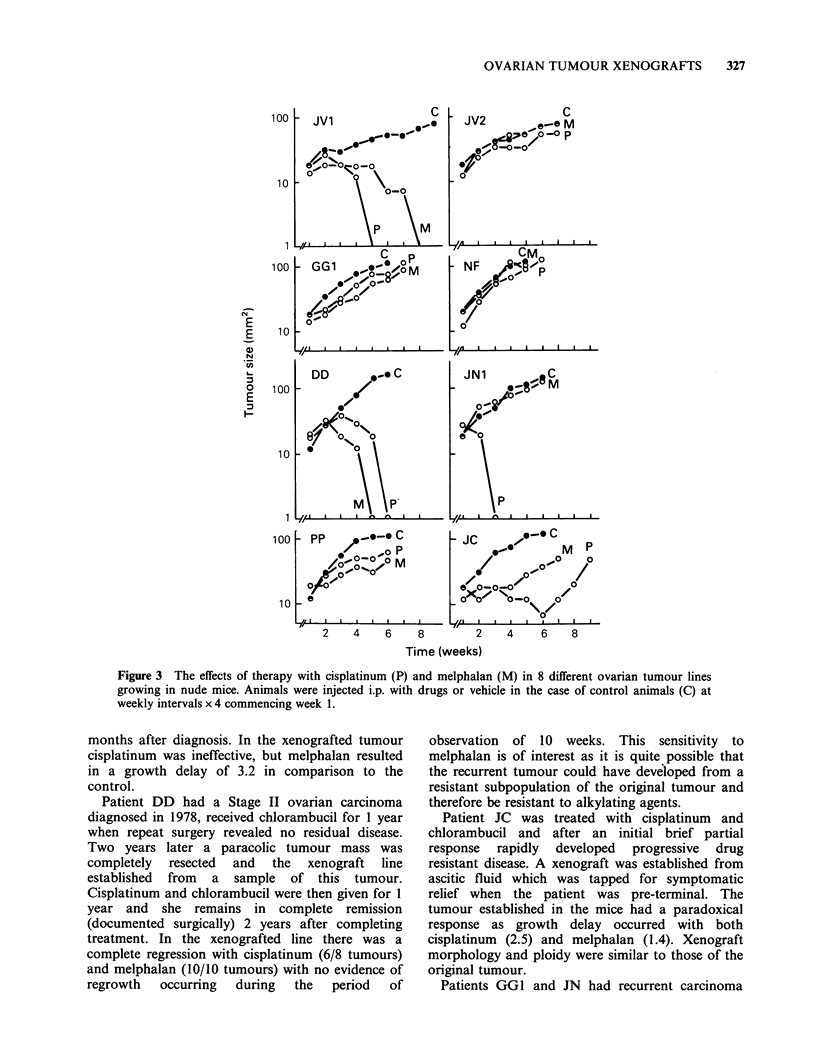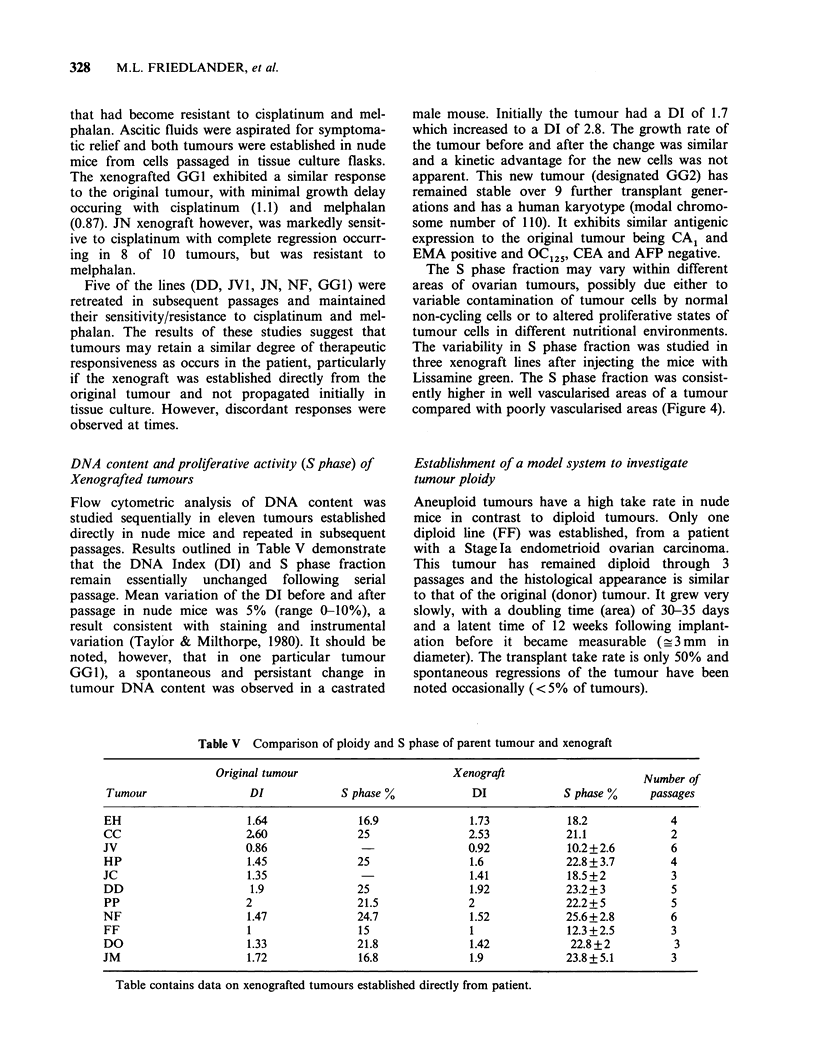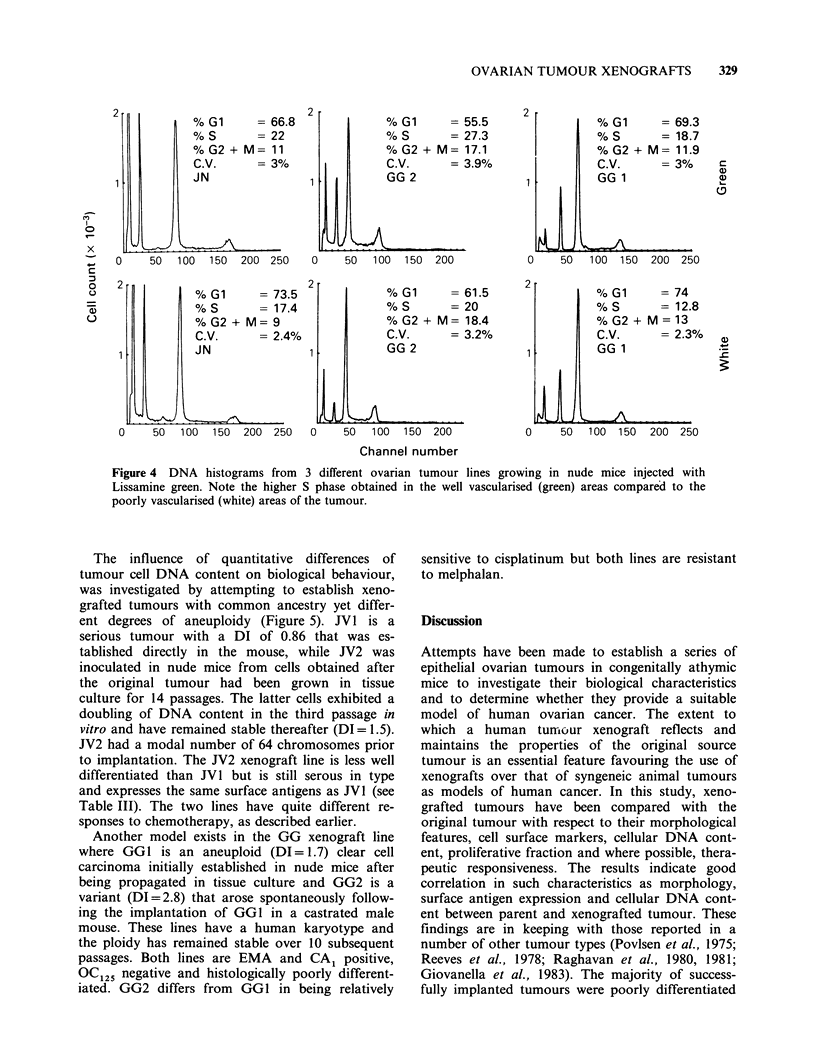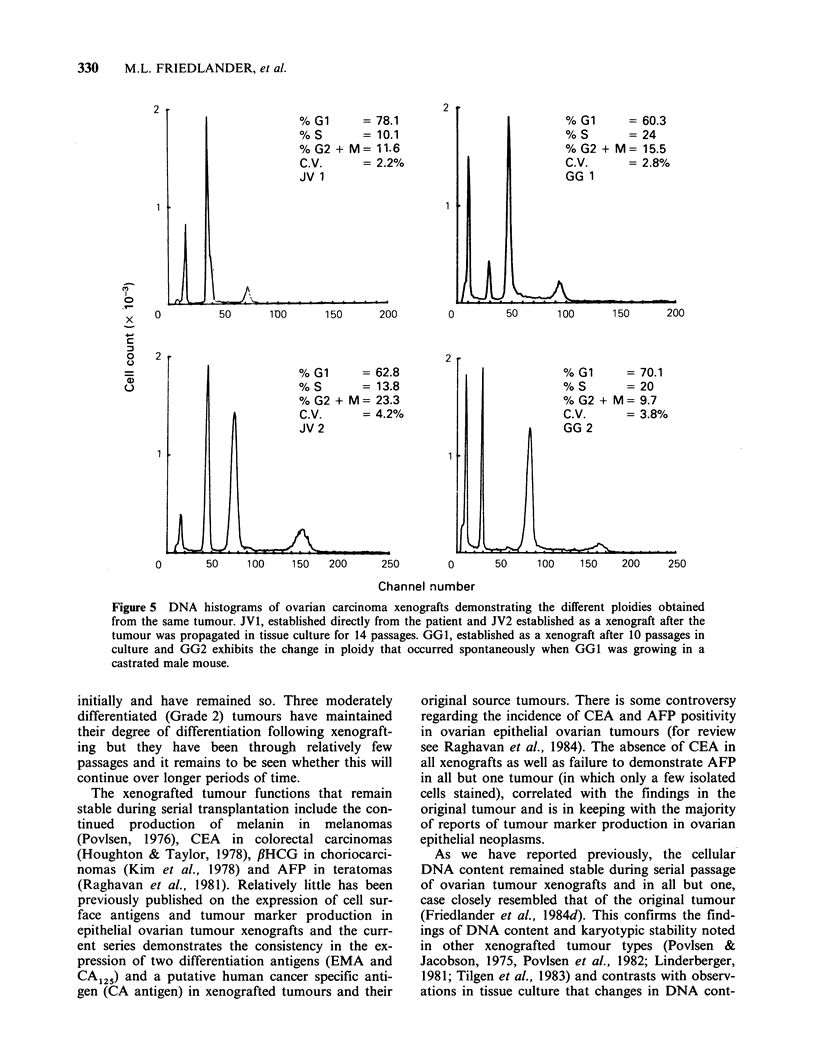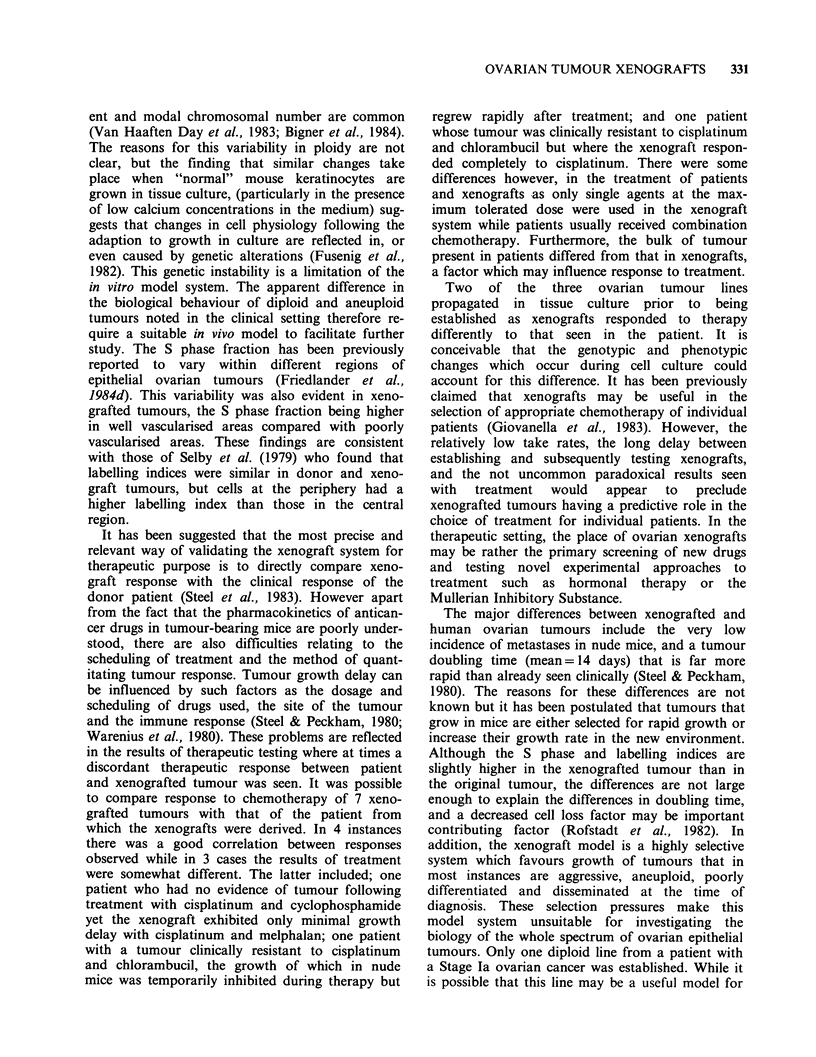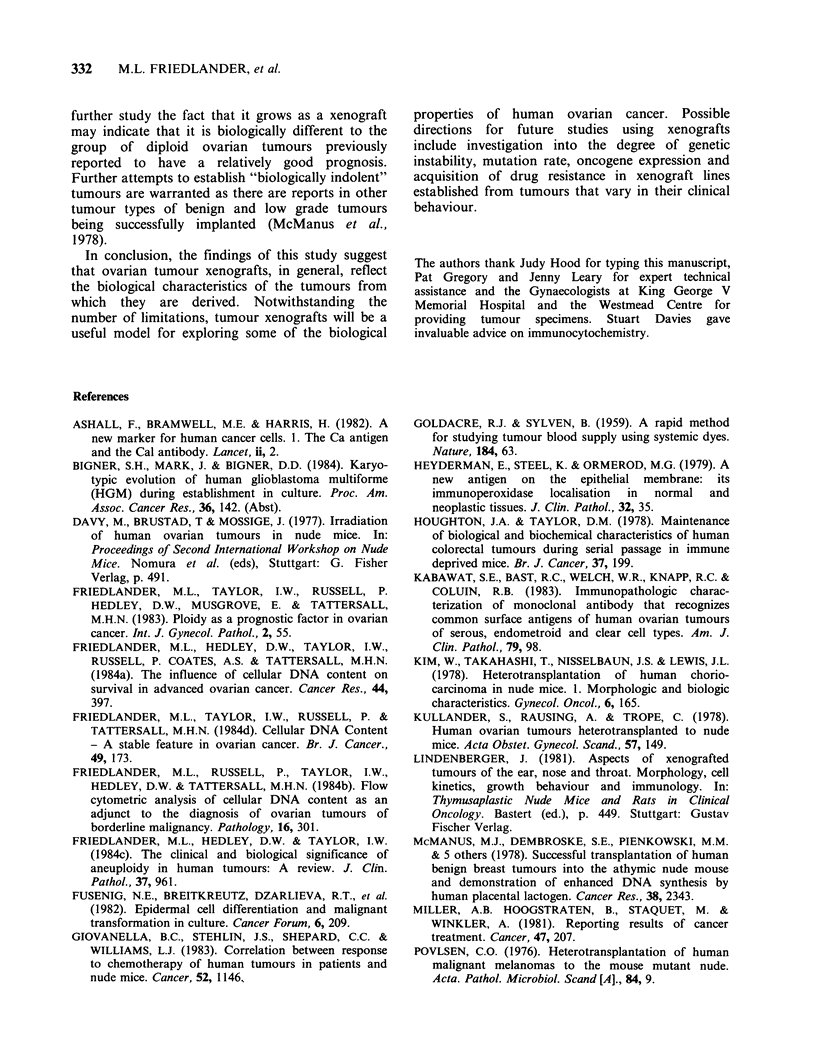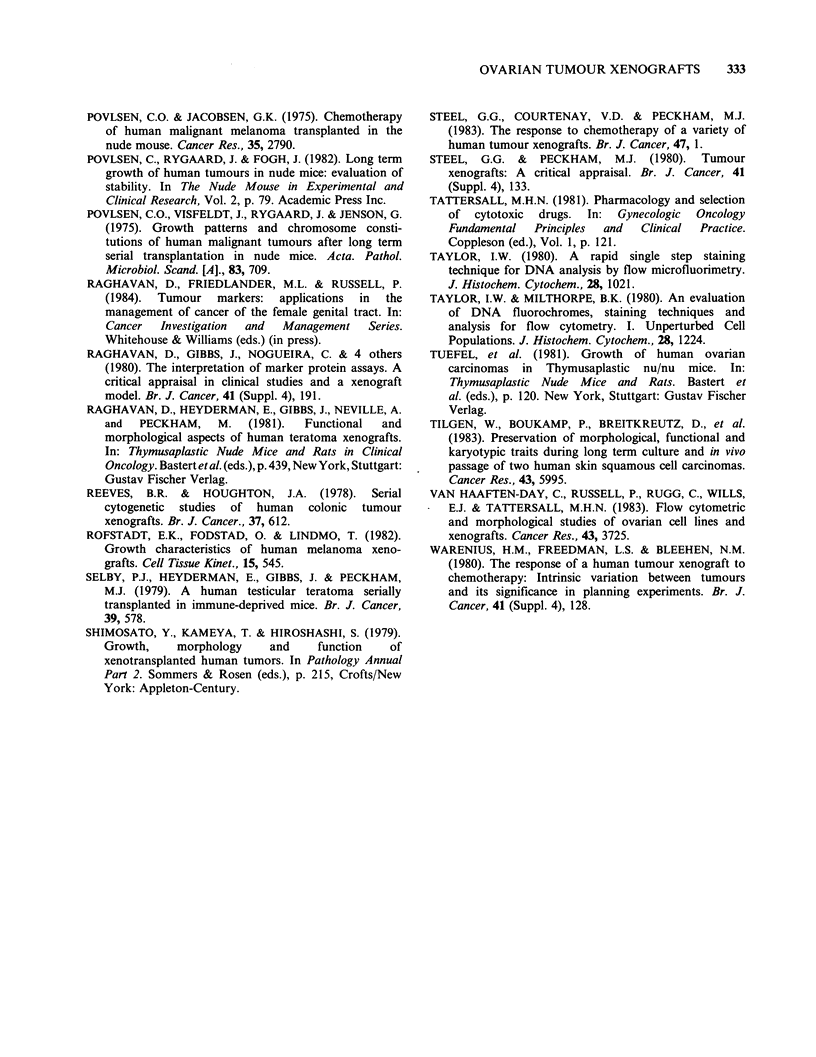# Ovarian tumour xenografts in the study of the biology of human epithelial ovarian cancer.

**DOI:** 10.1038/bjc.1985.44

**Published:** 1985-03

**Authors:** M. L. Friedlander, P. Russell, I. W. Taylor, M. H. Tattersall

## Abstract

**Images:**


*Br. J. Cancer* (1985), **51**, 319–333

### Ovarian tumour xenografts in the study of the biology of human epithelial ovarian cancer

M.L. Friedlander[1]*, P. Russell[2], I.W. Taylor[1]† & M.H.N. Tattersall[1]

[1]*Ludwig Institute for Cancer Research (Sydney Branch), Blackburn Building, University of Sydney, NSW 2006;* [2]*Department of Anatomical Pathology, King George V Memorial Hospital, Camperdown, NSW 2050, Australia.*

**Summary** Human epithelial ovarian tumours were successfully established as xenografts in nude mice in 54% of cases. An evaluation of the biological characteristics of tumours propagated in nude mice was carried out and the functions investigated included morphology, growth kinetics, cellular DNA content, cell surface antigen expression and sensitivity to chemotherapy. To allow a more detailed study of the influence of ploidy on biological behaviour, xenografted tumour with varying degrees of aneuploidy and tumours with a common ancestry but different ploidies were also established. Although this is a highly selective model system favouring the growth of biologically aggressive tumours the xenografts, in general, reflect many of the characteristics of the tumours from which they were derived and are likely to provide a useful model for investigating the biology of ovarian cancer.

The development of the congenitally athymic mouse mutant has provided a host that supports growth of human malignant tumours and offers unique possibilities to study the biology of human tumours *in vivo*. Of paramount importance in assessing the value of the xenograft model has been the evaluation of biological characteristics of tumours propagated in nude mice compared to those of the parent (donor) tumour. The general consensus reached in a number of studies encompassing a variety of tumour types is that human tumour xenografts largely reflect the features of the donor tumour with the exception of proliferation kinetics and metastatic properties (for reviews see Shimosata *et al.*, 1982; Povlsen *et al.*, 1982). However, there have been few detailed studies on the utility of ovarian tumour xenografts (Davy *et al.*, 1977; Kullander *et al.*, 1978; Teufel *et al.*, 1981; Van Haaften Day *et al.*, 1983). The aims of this study were two fold, firstly to investigate biological characteristics of ovarian tumour xenografts compared with those of the original tumour from which they were established, and secondly to establish a model system of xenografted tumours with different ploidies. Results of clinical flow cytometric studies have indicated that cellular DNA content is of prognostic importance in ovarian epithelial tumours (Friedlander *et al.*, 1984a, b) as well as in a number of other tumour types (for review see Friedlander *et al.*, 1984c) yet the fundamental reasons for this remain obscure. Establishment of diploid ovarian tumours together with tumours with varying degrees of aneuploidy in nude mice would allow more detailed study of the possible relationship between tumour ploidy and biological behaviour.

*Correspondence: M.L. Friedlander; Present address: Department of Medical Oncology, Princess Margaret Hospital, 500 Sherbourne Street, Toronto, Canada, M4X 1K9.

†Present address: Queensland Institute for Medical Research, Bramston Terrace, Herston, Brisbane, 4006.

Received 19 July 1984; and in revised form 14 November 1984.

### Materials and methods

### *Ovarian tumour xenografts*

*Establishment and maintenance of xenografts* Female BALB/c nude mice were obtained from the Australian Atomic Energy Commission, Lucas Heights, NSW, and housed in laminar flow racks and provided with sterilised food and drinking water *ad libitum*. Sterilised gloves, clean gowns, face masks and caps were used when handling the animals.

Tumour tissue was obtained directly from the operating theatre under sterile conditions, placed in RPMI 1640 medium containing 10% foetal calf serum and dissected into fragments measuring 3–5 mm in diameter. A sample was set aside for flow cytometry and histology. Xenografts were established in 6 week old mice by inoculating these fragments s.c., using fine forceps, through a small incision in the scapular region of the mouse. The incision was sutured with fine silk, a procedure which improved the take rate by ensuring fragments remained in place. Bilateral implantation was performed in all instances under ether

© The Macmillan Press Ltd., 1985

anaesthesia. Depending on the size of tumour sample obtained 3–5 mice were used in the attempt to establish xenografts.

When establishing xenografts from ascites or from *in vitro* cell lines, $\sim 5\times 10^7$ cells were injected s.c. in 0.1–0.2 ml of medium. The effusions were collected under sterile conditions, centrifuged at 200*g* for 10 min in 50 ml propylene centrifuge tubes (Corning Glass Company, Corning, NY) and the pellet resuspended in phosphate buffered saline (PBS) and washed twice prior to inoculation.

A tumour "take" was defined as a tumour which grew progressively after implantation and could be serially passaged. In the event of no growth occurring 8 months–1 year after implantation of the original tumour from the patient, the mice were sacrificed and this was defined as negative take.

When the tumours reached a size of 10–15 mm in diameter they were serially passaged by s.c. implantation of explants of xenografted tissue as described above. At this time samples of tissue were taken for flow cytometry and also fixed in combined formalin, acetic acid and alcohol (FAA) for subsequent histological examination. Autopsies were performed on most of these mice and specimens fixed in FAA for histological examination if there was macroscopic evidence of metastasis.

*Growth characteristics* Tumours were measured weekly, when they achieved a size of 3–5 mm in diameter, using graduated calipers. Growth curves were expressed as tumour size *versus* time, where the area ($mm^2$) was calculated by using the formula for an ellipse,

$$\pi + \frac{d1}{2} \times \frac{d2}{2}$$

with d1 and d2 representing the maximum diameters of the tumour measured at right angles (Teufel *et al.*, 1981).

Median tumour size was plotted against time and the doubling time (TD) found by interpolation on a semi-logarithmic plot (Steel *et al.*, 1983). The time taken for control and treated tumours to double in size (area) was determined and the difference (TD treated–TD control) was taken to approximate the growth delay. In order to allow comparison in delay values between tumour types that vary widely in growth rate, the specific growth delay was used to estimate the number of doubling times saved by treatment. (It should be noted that area doubling times are somewhat longer than volume doubling time.)

$$\text{specific growth delay} = \frac{\text{TD treated–TD control}}{\text{TD control}}$$

*Flow cytometry* Flow cytometric analysis of DNA content and proliferative fraction was performed, using a rapid staining technique previously described (Taylor, 1980; Friedlander *et al.*, 1983), on all primary tumour specimens, after most routine passages of "stock" tumours and on tumours implanted for chemosensitivity testing.

Regional heterogeneity with respect to ploidy and S phase fraction in xenografted tumours was investigated by sampling tissue from tumour areas with different vascularisation. The vascularity of tumours was determined by injecting mice i.p. with 0.2–0.3 ml of a 2% solution of Lissamine green. This non-toxic dye colours the animal green in about an hour and the colour begins to fade in a few hours. Lissamine green delineates various areas within a tumour with the well vascularised regions staining deep green and avascular regions remaining white (Goldacre & Sylven, 1959). Tumour samples/fine needle aspirates from differently staining areas were then taken and prepared for flow cytometric analysis.

*Chemotherapy* A series of experiments was conducted to study the chemosensitivity of xenografted tumour and compared with the results of treatment in the donor patient. Standard criteria for response were used for assessing the response to treatment in patients (Miller *et al.*, 1981).

Single agent chemotherapy was given by intraperitoneal injection, the therapeutic dosage being determined in a series of toxicity experiments by treating groups of 5 mice with varying dose levels. The maximum tolerated dose (MTD) was used and defined as the drug dose that caused <20% decrease in body weight and did not kill any mice.

The final doses used in chemosensitivity testing were as follows:

Cisplatinum 5 mg kg$^{-1}$ i.p. weekly × 4.

Melphalan 5 mg kg$^{-1}$ i.p. weekly × 4.

Drugs were freshly made up for each experiment. Control animals were treated with normal saline (in the case of cisplatinum treatment) or with melphalan diluent (in the case of melphalan therapy) in an equivalent volume, and control and test tumour growth curves were compared. In the majority of experiments there were 5 animals in the treated and control groups. Tumours were measured weekly using calipers, as described earlier.

### *Histology*

Specimens for histological examination were taken from the tumours destined for implantation and also from the resulting xenograft. Tissues were routinely fixed in FAA both for cytomorphology and immunocytochemical staining.

Immunocytochemical staining was performed using the immunoperoxidase technique described. The antigens investigated included; CA, a putative cancer specific antigen (Ashall *et al.*, 1982); $CA_{125}$, a Mullerian differentiation antigen (Kabawat *et al.*, 1983); EMA, an epithelial membrane antigen (Heyderman *et al.*, 1979); and the oncofoetal antigens alphafoetoprotein (AFP) and carcinoembryonic antigen (CEA).

*Immunocytochemical techniques* The basic immunoperoxidase technique is outlined below with modifications according to the particular antibody used.

Steps:

1. Dewax 7$\mu$m paraffin section in xylene.
2. Remove xylene in absolute ethanol (3 changes of 2 min each).
3. Block endogenous peroxidase activity by incubating sections in 0.3% hydrogen peroxide in methanol for 20 min.
4. Rinse in tap water followed by Tris buffered saline (pH 7.6).
5. Stand in non-immune swine serum for at least 2 min to reduce background staining.
6. Incubate sections in primary antibody for 1 h (see below for details).
7. Wash slides in Tris buffer (pH 7.6) × 3.
8. Rinse quickly in non-immune swine serum.
9. Incubate sections with secondary antibody (usually swine anti-rabbit immunoglobin 1:50 dilution) for 15 min.
10. Wash slides in Tris buffer (pH 7.6) × 3.
11. Rinse quickly in non-immune swine serum.
12. Incubate sections for 15 min at room temperature with PAP complex raised in rabbit, diluted 1:100.
13. Rinse × 3 with Tris buffer.
14. Cover with freshly prepared diaminobenzidine (DAB), to develop peroxidase reaction, for 10 min (DAB 40 mg % in phosphate buffer pH 6.8 with 25 $\mu$l $H_2O_2$/100 ml of buffer added immediately prior to use).
15. Wash well in tap water.
16. Counterstain with haematoxylin for 5 min.
17. Rinse in water.
18. Differentiate in Acid Alcohol (1% HCL in 70% alcohol).
19. Blue in running warm water.
20. Dehydrate through ethanol and mount.

*Modifications according to antibody used*

*$CA_1$* The $CA_1$ monoclonal antibody (Wellcome diagnostics) was purchased as freeze dried residue of 2 ml of tissue culture fluid containing 25 $\mu$g of $CA_1$ antibody, 10% foetal calf serum and 0.1% sodium azide as preservative. Sections were incubated in the primary anti $CA_1$ antibody (dilution 1:100) for 16 h at 4°C (Step 6). This was followed by an additional step-exposure to rabbit anti-mouse IgM (Miles Laboratories) at a dilution of 1:500 for 15 min. The rest of the procedure from Step 7 was carried out as outlined above.

*$OC_{125}$* $OC_{125}$ is a mouse $IgG_1$ monoclonal antibody that was a gift from Dr R.C. Bast (Dana Faber Cancer Institute, Boston). The method used by Bast *et al.* (1983) to demonstrate the $CA_{125}$ antigen was an indirect immunofluorescence technique on cryostat sections of ovarian tissue (Kabawat *et al.*, 1983). The technique was modified to allow detection of the $CA_{125}$ antigen in FAA or formalin-fixed, paraffin-embedded tissues. Sections were incubated in $OC_{125}$ antibody at a dilution of 1:100 for 16 h at 4°C (Step 6). This was followed by an additional linking step by exposure of sections to rabbit anti-mouse immunoglobulin (DAKO) diluted 1:5000 for 15 min. The rest of the procedure from Step 7 onwards was performed as outlined above.

*Carcinoembryonic antigen: (CEA)* Spleen absorbed CEA (DAKO) was used at a dilution of 1:500 (Step 6).

*Alphafoetoprotein: (AFP)* AFP (DAKO) was used at a dilution of 1:500 (Step 6).

*Epithelial membrane antigen: (EMA)* Sections were incubated in goat EMA (SERA-LAB) at a dilution of 1:500 for 16 h at 4°C (Step 6). This was followed by a linking step using rabbit anti-goat (DAKO) at a dilution of 1:100. The rest of the procedure is outlined from Step 7 onwards, except that goat PAP (DAKO) was used instead of rabbit PAP (Step 12).

*Controls*

Known positive and negative tissue sections were included to exclude false positives and negatives which could arise due to technical problems. Negative controls for $OC_{125}$ included incubating slides with T11, a purified mouse $IgG_1$ pan T cell antibody and also by omitting $OC_{125}$ from the staining procedure. In the ovarian xenografts the primary monoclonal antibody was also omitted and the sections incubated in the presence of the rabbit anti-mouse antibody alone to confirm that there was no nonspecific staining of tumour tissue.

### Results

### *Tumourigenicity of ovarian tumours in nude mice*

Twenty-three epithelial ovarian tumours and 3 cultured ovarian carcinoma lines were implanted in nude mice and progressive growth was evident in 16 cases (67%). Fourteen tumours were serially passaged giving an overall successful take rate of 54%. Table I outlines details of the donor tumours, that were implanted directly from the patient. Advanced stage tumours had a higher likelihood of a successful tumour take compared with early stage (F.I.G.O. Stages I and II) tumours ($P=0.01$; $\chi^2$ 9.64). The advanced tumours (F.I.G.O. Stage III) were invariably aneuploid, usually poorly differentiated and in four instances tumours were obtained from patients at the time of recurrence. In contrast, borderline tumours and early stage malignant ovarian cancers were associated with a low likelihood of being successfully transplanted and were commonly diploid (Table I). There also appeared to be a relationship between tumour take and patient survival: 60% of patients associated with a successful tumour take died of ovarian cancer, compared with 10% of patients where the tumour did not take.

### *Growth of ovarian tumours in nude mice*

The mean delay from initial implantation to the first passage was 14 weeks (range 5–36 weeks) (Table II). The time between subsequent passages of stock tumours was quite variable and depended predominantly on the size of tumour fragments that were initially implanted and the size of the stock tumours attained when the mice were sacrificed. In general, 2 mm x 2 mm tumour fragments were implanted and the tumours passaged when they reached a size of 10–15 mm in diameter.

Growth rates of the xenografted tumours were determined by weekly caliper measurements of groups of 8–10 tumours. Mean area (size) doubling time (TD) was determined in 10 xenograft lines to be 14 days (range 5–35 days). The mean take rate

**Table I** Details of ovarian tumours implanted directly in nude mice

| *Tumour* | *Stage* | *Type* | *Grade* | *Ploidy (DI)* | *S phase (%)* | *Take* | *Patient survival (Weeks)* |
|---|---|---|---|---|---|---|---|
| VB[a] | 1 | E | 3 | 1 | 11.6 | – | 140A |
| JL | 1 | E | 1 | 1 | 5 | – | 20A |
| JH | 1 | M (LPM) | | 1 | 8.9 | – | 44A |
| RL | 1 | M (LPM) | | 1 | 5.5 | – | 40A |
| SG | 1 | S | | 1 | 4.3 | – | 60A |
| FF | 1 | E | 2 | 1 | 15 | + | 80A |
| EB | 1 | C | – | 3.3 | — | – | 72A |
| MC | 1 | E | 3 | 1 | 7.9 | – | 80A |
| MA | 1 (REC) | S | 1 | 1.1 | — | – | 50A |
| AM[a] | 1 | CS | – | 1.68 | 18.5 | +[b] | 104A |
| SH | 2 | E | 2 | 1.2 | — | – | 72A |
| PP | 2 | E | 3 | 2 | 21.5 | + | 72A |
| DD[a] | 2 (REC) | S | 3 | 1.9 | 25 | + | 156A |
| JM | 3 | S | 3 | 1.72 | 16.8 | + | 75A |
| CC | 3 | S | 3 | 2.60 | 25 | + | 72D |
| JC | 3 (REC) | S | 3 | 1.35 | — | + | 1D |
| EH[a] | 3 | S | 3 | 1.64 | 16.9 | + | 26D |
| JV[a] | 3 | S | 2 | 0.86 | — | + | 56D |
| NW[a] | 3 | S | 3 | 0.8 | — | +[b] | 70D |
| NF[a] | 3 | CS | – | 1.47 | 24.7 | + | 12D |
| HP[a] | 3 | S | 3 | 1.45 | 25 | + | 52LTF |
| DO | 3 | S | 2 | 1.33 | 21.8 | + | 1D[c] |
| BR | 3 (REC) | S | 3 | 1.53 | — | – | 20D |

S = serous; E = endometrioid; M = mucinous; C = clear cell; CS = carcinosarcoma; LPM = low potential malignancy; REC = recurrent tumour.

[a]Tumours implanted at LICR by C. Van Haaften Day; A = alive; D = dead; LTF = lost to follow up.

[b]Failed to grow in subsequent passage.

[c]Post operative death. Table does not include the 3 tumours estimated from ovarian carcinoma cells in culture (GG, JN, JV2). DI = DNA index, this represents the ratio of the DNA content of tumour $G_1$ cells to the diploid $G_1$ peak.

**Table II** Time between initial implantation and subsequent passages, the take rate and tumour doubling time in xenografted lines

| *Tumour* | *Time from primary implant to P1 (weeks)* | *Time between subsequent passages (weeks)* | *Take rate %* | *TD (days)* |
|---|---|---|---|---|
| PP | 5 | 8–12 | 83 | 5 (P2) |
| DD | 10 | 12–16 | 80 | 7 (P6) |
| JN | P12[a] | 6–12 | >90 | 5 (P8) |
| JC | 9 | 8–12 | 90 | 9 (P2) |
| JV1 | 26 | 12–14 | 80 | 21 (P6) |
| JV2 | 12[a] | 12 | 70 | 28 (P1) |
| NF | 6 | 4–4 | >90 | 7 (P4) |
| GG1 | 20[a] | 12–20 | >90 | 8 (P4) |
| GG2 | — | 8–18 | >90 | 7 (P11) |
| FF | 16 | 24 | 50 | 35 (P2) |
| DO | 12 | 12–24 | ND | ND |
| JM | 12 | 16 | ND | ND |
| CC | 12 | >12 | ND | ND |
| JP | 20 | 14–24 | ND | ND |
| EH | 29 | 16–20 | ND | ND |

[a]Established from tissue culture; ND = not determined – tumours maintained in 2 stock animals; (P) = passage in which doubling time, TD, determined. Take refers to the number of tumours that grew progressively and the % take in the Table refers to the mean % take for each particular tumour for all the passages it has been through.

of these lines in subsequent passages was 81% with the lowest take rate of 50% occurring in the diploid line (Table II).

### *Morphology and function of ovarian xenografts*

Transplanted tumours grew locally at the site of implantation and were strictly confined to the subcutaneous space. They did not invade surrounding soft tissues and could be readily dissected from the adjacent dermis and fascia. Metastatic spread was observed in only one slowly growing tumour (DO) which metastasised to the lungs during its first passage. The pulmonary metastases closely resembled the histopathological features of the original tumour. Whether this is a consistent feature of this particular tumour is not known as yet and is being investigated.

The majority of tumours grew as solid nodules, but in some cases there were large areas of cystic degeneration noted. The degree of vascularity varied among tumour types with some tumours being relatively avascular while others showed prominent neovascularisation mainly from axillary vessels. Haemorhagic necrosis occurred when such tumours grew beyond 10–15 mm in diameter.

The cytomorphology of the original tumours were usually maintained following xenotransplantation although there were often minor differences in architecture presumably due to a different stromal reaction. There was no evidence of increased differentiation occurring in any of the tumours, the majority of the original tumours were poorly differentiated (Grade III) and remained so in subsequent passages. Three tumours (FF, JV, DO) were moderately well differentiated when initially implanted and have maintained their morphology (Figure 1a, b), but they have been through only few passages to date. In contrast those tumours which had been initially established in cell culture and passaged up to 14 times before being implanted became progressively less differentiated following xenografting (Figure 1c).

### *Expression of cell surface antigens*

The expression of a number of antigenic determinants was compared in xenografted tumours and the corresponding original (donor) tumour where adequate well fixed tissue was available for immunocytochemistry. Antigens investigated included the epithelial membrane antigen (EMA), the CA antigen, the $CA_{125}$ antigen and the oncofoetal antigens, alphafoetoprotein (AFP) and carcinoembryonic antigen (CEA). The results of the study are outlined in Table III and disclose no appreciable differences in the expression of these antigens between the xenografted and donor tumours (Figure 2). The patterns and distribution of staining of EMA, $CA_1$ and $OC_{125}$ were similar in the xenografted tumour tissue although, there was a greater degree of cytoplasmic staining present,

**Figure 1** (a) Morphology of moderately differentiated serous carcinoma (JV) before xenografting ( × 250); (b) Morphology of moderately differentiated serous carcinoma after xenografting (JV1, passage 7) ( × 250); (c) Morphology of JV2 xenograft (P1). This was established from a cell line (OC: JV2) obtained by growing the original JV tumour in tissue culture. Undifferentiated carcinoma exhibiting cell to cell variation and nuclear anaplasia. This tumour became more aneuploid during cell culture and had a modal chromosome number of 64.

**Table III** Expression of cell surface antigens in the original and xenografted ovarian tumours

| | | | $CA_1$ | | $OC_{125}$ | | $EMA$ | | $CEA$ | | $AFP$ | |
|---|---|---|---|---|---|---|---|---|---|---|---|---|
| *Tumour* | *Passage* | *Histology* | *PT* | *XN* | *PT* | *XN* | *PT* | *XN* | *PT* | *XN* | *PT* | *XN* |
| JV1 | 6 | Serous | + | + | + | + | + | + | − | − | − | − |
| JV2[a] | 1 | Serous | + | + | + | + | + | + | − | − | − | −[b] |
| JN | 5 | Serous | ND | + | ND | + | ND | + | − | − | − | − |
| JC | 1 | Serous | + | + | + | + | + | + | − | − | − | − |
| EH | 2 | Serous | + | + | + | + | + | + | − | − | − | − |
| CG[a] | 7 | Clear cell | + | + | + | − | + | + | − | − | − | − |
| JM | 1 | Serous | + | + | + | + | + | + | − | − | − | − |
| DO | 1 | Serous | ± | ± | + | + | + | + | − | − | − | − |
| PP | 1 | Adenosquamous | − | − | − | − | +[b] | +[b] | − | − | ±[b] | ±[b] |
| JP | 1 | Serous | ND | + | ND | + | ND | + | − | − | − | − |
| FF | 1 | Endometrioid | − | ± | + | + | + | + | − | − | − | − |
| NF | 1 | Carcinosarcoma | − | − | − | − | − | − | − | − | − | − |
| DD | 6 | Serous | − | − | − | − | + | + | − | − | − | − |

+ = > 10% of cells stain positively; ± = < 10% of cells stain; − = no detectable staining.
[a]Established from cultured line; [b] = adenocarcinoma cells only stain; ND = not done; PT = donor tumour; XN = xenografted tumour.

particularly for $CA_1$. In all tumours studied, apart from one carcinosarcoma, both the parent and xenografted tumour were EMA positive. A feature of most tumours analysed for reactivity with $OC_{125}$ and $CA_1$ was a variability in staining between otherwise morphologically indistinguishable cells. This heterogeneity in cell surface antigen expression was maintained in the xenografted tumours. The expression of CEA and AFP was also compared in the xenografted and respective original tumours to investigate their stability. However, apart from a single case where AFP could be demonstrated in a

**Figure 2** Comparison of EMA staining in (a) original serous carcinoma and (b) after xenografting ( × 200).

few scattered cells in both the original and xenografted tumour (PP), they were all CEA and AFP negative.

*The* in Situ *response of xenografts to chemotherapy*

The responses of 8 ovarian xenografted tumour lines to treatment with cisplatinum and melphalan are shown in Table IV and Figure 3. Although chlorambucil was used in patients, melphalan was chosen in preference to treat the xenografts because of the availability of a commercial preparation suited for i.p. injection. The mechanisms of action of melphalan and chlorambucil are similar and comparable response rates for each drug have been reported in ovarian cancer (for review, see Tattersall, 1981).

Patient JV had an unresectable Stage III serious carcinoma and was treated with a combination of cisplatinum and chlorambucil with a good partial response and minimal residual pelvic tumour was clinically evident after 4 months of therapy. She relapsed however, 3 months later while still on treatment and eventually died with drug resistant disease. The JV1 line, established directly from this patient, responded completely to cisplatinum and melphalan with no evidence of regrowth occurring during a 6 week period of observation. In contrast, the JV2 line which was established in the xenograft only after being propagated in tissue culture did not respond to cisplatinum or melphalan. This carcinoma was histologically less well differentiated than JV1 and has approximately twice its cellular DNA content.

Patient NF had an extremely aggressive and widely metastatic carcinosarcoma which did not respond to a combination of chlorambucil and provera and the patient died with progressive disease within 3 months of diagnosis. The xenografted tumour grew rapidly and was totally resistant to cisplatinum and melphalan.

Patient PP had a Stage IIc adenosquamous carcinoma that was completely resected and she then received a combination of cisplatinum for 8 courses together with cyclophosphamide for 1 year. She remains well and with no evidence of disease 18

**Table IV** Effect of chemotherapy on ovarian tumour xenografts

| *Tumour* | *Treatment* | *Passage treated* | *T D (days)* | *Growth delay* |
|---|---|---|---|---|
| DD+ | Control | P6 | 7 | |
| | Cisplatinum | | 6/8 CR | |
| | Melphalan | | 10/10 CR | |
| PP | Control | P2 | 5 | |
| | Cisplatinum | | 5 | 0 |
| | Melphalan | | 21 | 3.2 |
| JN[a]+ | Control | P8 | 5 | |
| | Cisplatinum | | 8/10 CR | |
| | Melphalan | | 5 | 0 |
| JC+ | Control | P2 | 9 | |
| | Cisplatinum | | 32 | 2.5 |
| | Melphalan | | 22 | 1.4 |
| JV1 | Control | P6 | 21 | |
| | Cisplatinum | | 9/10 CR | |
| | Melphalan | | 7/10 CR | |
| JV2[a] | Control | P1 | 28 | |
| | Cisplatinum | | 24.5 | |
| | Melphalan | | 28 | 0 |
| NF | Control | P4 | 7 | |
| | Cisplatinum | | 6 | 0 |
| | Nelphalan | | 7 | 0 |
| GG1[a]+ | Control | P4 | 8 | |
| | Cisplatinum | | 17 | 1.1 |
| | Melphalan | | 16 | 0.875 |

CR = complete regression, indicates complete disappearance of a tumour for a period of observation of up to 10 weeks, at which time the mice were sacrificed.

[a]Xenograft established from cell line; + = xenograft established from recurrent tumour.

**Figure 3** The effects of therapy with cisplatinum (P) and melphalan (M) in 8 different ovarian tumour lines growing in nude mice. Animals were injected i.p. with drugs or vehicle in the case of control animals (C) at weekly intervals x 4 commencing week 1.

months after diagnosis. In the xenografted tumour cisplatinum was ineffective, but melphalan resulted in a growth delay of 3.2 in comparison to the control.

Patient DD had a Stage II ovarian carcinoma diagnosed in 1978, received chlorambucil for 1 year when repeat surgery revealed no residual disease. Two years later a paracolic tumour mass was completely resected and the xenograft line established from a sample of this tumour. Cisplatinum and chlorambucil were then given for 1 year and she remains in complete remission (documented surgically) 2 years after completing treatment. In the xenografted line there was a complete regression with cisplatinum (6/8 tumours) and melphalan (10/10 tumours) with no evidence of regrowth occurring during the period of observation of 10 weeks. This sensitivity to melphalan is of interest as it is quite possible that the recurrent tumour could have developed from a resistant subpopulation of the original tumour and therefore be resistant to alkylating agents.

Patient JC was treated with cisplatinum and chlorambucil and after an initial brief partial response rapidly developed progressive drug resistant disease. A xenograft was established from ascitic fluid which was tapped for symptomatic relief when the patient was pre-terminal. The tumour established in the mice had a paradoxical response as growth delay occurred with both cisplatinum (2.5) and melphalan (1.4). Xenograft morphology and ploidy were similar to those of the original tumour.

Patients GG1 and JN had recurrent carcinoma that had become resistant to cisplatinum and melphalan. Ascitic fluids were aspirated for symptomatic relief and both tumours were established in nude mice from cells passaged in tissue culture flasks. The xenografted GG1 exhibited a similar response to the original tumour, with minimal growth delay occuring with cisplatinum (1.1) and melphalan (0.87). JN xenograft however, was markedly sensitive to cisplatinum with complete regression occurring in 8 of 10 tumours, but was resistant to melphalan.

Five of the lines (DD, JV1, JN, NF, GG1) were retreated in subsequent passages and maintained their sensitivity/resistance to cisplatinum and melphalan. The results of these studies suggest that tumours may retain a similar degree of therapeutic responsiveness as occurs in the patient, particularly if the xenograft was established directly from the original tumour and not propagated initially in tissue culture. However, discordant responses were observed at times.

### *DNA content and proliferative activity (S phase) of Xenografted tumours*

Flow cytometric analysis of DNA content was studied sequentially in eleven tumours established directly in nude mice and repeated in subsequent passages. Results outlined in Table V demonstrate that the DNA Index (DI) and S phase fraction remain essentially unchanged following serial passage. Mean variation of the DI before and after passage in nude mice was 5% (range 0–10%), a result consistent with staining and instrumental variation (Taylor & Milthorpe, 1980). It should be noted, however, that in one particular tumour GG1), a spontaneous and persistant change in tumour DNA content was observed in a castrated male mouse. Initially the tumour had a DI of 1.7 which increased to a DI of 2.8. The growth rate of the tumour before and after the change was similar and a kinetic advantage for the new cells was not apparent. This new tumour (designated GG2) has remained stable over 9 further transplant generations and has a human karyotype (modal chromosome number of 110). It exhibits similar antigenic expression to the original tumour being $CA_1$ and EMA positive and $OC_{125}$, CEA and AFP negative.

The S phase fraction may vary within different areas of ovarian tumours, possibly due either to variable contamination of tumour cells by normal non-cycling cells or to altered proliferative states of tumour cells in different nutritional environments. The variability in S phase fraction was studied in three xenograft lines after injecting the mice with Lissamine green. The S phase fraction was consistently higher in well vascularised areas of a tumour compared with poorly vascularised areas (Figure 4).

### *Establishment of a model system to investigate tumour ploidy*

Aneuploid tumours have a high take rate in nude mice in contrast to diploid tumours. Only one diploid line (FF) was established, from a patient with a Stage Ia endometrioid ovarian carcinoma. This tumour has remained diploid through 3 passages and the histological appearance is similar to that of the original (donor) tumour. It grew very slowly, with a doubling time (area) of 30–35 days and a latent time of 12 weeks following implantation before it became measurable ($\cong$3 mm in diameter). The transplant take rate is only 50% and spontaneous regressions of the tumour have been noted occasionally (<5% of tumours).

**Table V** Comparison of ploidy and S phase of parent tumour and xenograft

| *Tumour* | *Original tumour* *DI* | *S phase %* | *Xenograft* DI | *S phase %* | *Number of passages* |
|---|---|---|---|---|---|
| EH | 1.64 | 16.9 | 1.73 | 18.2 | 4 |
| CC | 2.60 | 25 | 2.53 | 21.1 | 2 |
| JV | 0.86 | — | 0.92 | $10.2 \pm 2.6$ | 6 |
| HP | 1.45 | 25 | 1.6 | $22.8 \pm 3.7$ | 4 |
| JC | 1.35 | — | 1.41 | $18.5 \pm 2$ | 3 |
| DD | 1.9 | 25 | 1.92 | $23.2 \pm 3$ | 5 |
| PP | 2 | 21.5 | 2 | $22.2 \pm 5$ | 5 |
| NF | 1.47 | 24.7 | 1.52 | $25.6 \pm 2.8$ | 6 |
| FF | 1 | 15 | 1 | $12.3 \pm 2.5$ | 3 |
| DO | 1.33 | 21.8 | 1.42 | $22.8 \pm 2$ | 3 |
| JM | 1.72 | 16.8 | 1.9 | $23.8 \pm 5.1$ | 3 |

Table contains data on xenografted tumours established directly from patient.

**Figure 4** DNA histograms from 3 different ovarian tumour lines growing in nude mice injected with Lissamine green. Note the higher S phase obtained in the well vascularised (green) areas compared to the poorly vascularised (white) areas of the tumour.

The influence of quantitative differences of tumour cell DNA content on biological behaviour, was investigated by attempting to establish xenografted tumours with common ancestry yet different degrees of aneuploidy (Figure 5). JV1 is a serious tumour with a DI of 0.86 that was established directly in the mouse, while JV2 was inoculated in nude mice from cells obtained after the original tumour had been grown in tissue culture for 14 passages. The latter cells exhibited a doubling of DNA content in the third passage *in vitro* and have remained stable thereafter (DI = 1.5). JV2 had a modal number of 64 chromosomes prior to implantation. The JV2 xenograft line is less well differentiated than JV1 but is still serous in type and expresses the same surface antigens as JV1 (see Table III). The two lines have quite different responses to chemotherapy, as described earlier.

Another model exists in the GG xenograft line where GG1 is an aneuploid (DI = 1.7) clear cell carcinoma initially established in nude mice after being propagated in tissue culture and GG2 is a variant (DI = 2.8) that arose spontaneously following the implantation of GG1 in a castrated male mouse. These lines have a human karyotype and the ploidy has remained stable over 10 subsequent passages. Both lines are EMA and $CA_1$ positive, $OC_{125}$ negative and histologically poorly differentiated. GG2 differs from GG1 in being relatively sensitive to cisplatinum but both lines are resistant to melphalan.

### Discussion

Attempts have been made to establish a series of epithelial ovarian tumours in congenitally athymic mice to investigate their biological characteristics and to determine whether they provide a suitable model of human ovarian cancer. The extent to which a human tumour xenograft reflects and maintains the properties of the original source tumour is an essential feature favouring the use of xenografts over that of syngeneic animal tumours as models of human cancer. In this study, xenografted tumours have been compared with the original tumour with respect to their morphological features, cell surface markers, cellular DNA content, proliferative fraction and where possible, therapeutic responsiveness. The results indicate good correlation in such characteristics as morphology, surface antigen expression and cellular DNA content between parent and xenografted tumour. These findings are in keeping with those reported in a number of other tumour types (Povlsen *et al.*, 1975; Reeves *et al.*, 1978; Raghavan *et al.*, 1980, 1981; Giovanella *et al.*, 1983). The majority of successfully implanted tumours were poorly differentiated

**Figure 5** DNA histograms of ovarian carcinoma xenografts demonstrating the different ploidies obtained from the same tumour. JV1, established directly from the patient and JV2 established as a xenograft after the tumour was propagated in tissue culture for 14 passages. GG1, established as a xenograft after 10 passages in culture and GG2 exhibits the change in ploidy that occurred spontaneously when GG1 was growing in a castrated male mouse.

initially and have remained so. Three moderately differentiated (Grade 2) tumours have maintained their degree of differentiation following xenografting but they have been through relatively few passages and it remains to be seen whether this will continue over longer periods of time.

The xenografted tumour functions that remain stable during serial transplantation include the continued production of melanin in melanomas (Povlsen, 1976), CEA in colorectal carcinomas (Houghton & Taylor, 1978), βHCG in choriocarcinomas (Kim *et al.*, 1978) and AFP in teratomas (Raghavan *et al.*, 1981). Relatively little has been previously published on the expression of cell surface antigens and tumour marker production in epithelial ovarian tumour xenografts and the current series demonstrates the consistency in the expression of two differentiation antigens (EMA and $CA_{125}$) and a putative human cancer specific antigen (CA antigen) in xenografted tumours and their original source tumours. There is some controversy regarding the incidence of CEA and AFP positivity in ovarian epithelial ovarian tumours (for review see Raghavan *et al.*, 1984). The absence of CEA in all xenografts as well as failure to demonstrate AFP in all but one tumour (in which only a few isolated cells stained), correlated with the findings in the original tumour and is in keeping with the majority of reports of tumour marker production in ovarian epithelial neoplasms.

As we have reported previously, the cellular DNA content remained stable during serial passage of ovarian tumour xenografts and in all but one, case closely resembled that of the original tumour (Friedlander *et al.*, 1984*d*). This confirms the findings of DNA content and karyotypic stability noted in other xenografted tumour types (Povlsen & Jacobson, 1975, Povlsen *et al.*, 1982; Linderberger, 1981; Tilgen *et al.*, 1983) and contrasts with observations in tissue culture that changes in DNA content and modal chromosomal number are common (Van Haaften Day *et al.*, 1983; Bigner *et al.*, 1984). The reasons for this variability in ploidy are not clear, but the finding that similar changes take place when "normal" mouse keratinocytes are grown in tissue culture, (particularly in the presence of low calcium concentrations in the medium) suggests that changes in cell physiology following the adaption to growth in culture are reflected in, or even caused by genetic alterations (Fusenig *et al.*, 1982). This genetic instability is a limitation of the *in vitro* model system. The apparent difference in the biological behaviour of diploid and aneuploid tumours noted in the clinical setting therefore require a suitable *in vivo* model to facilitate further study. The S phase fraction has been previously reported to vary within different regions of epithelial ovarian tumours (Friedlander *et al., 1984d*). This variability was also evident in xenografted tumours, the S phase fraction being higher in well vascularised areas compared with poorly vascularised areas. These findings are consistent with those of Selby *et al.* (1979) who found that labelling indices were similar in donor and xenograft tumours, but cells at the periphery had a higher labelling index than those in the central region.

It has been suggested that the most precise and relevant way of validating the xenograft system for therapeutic purpose is to directly compare xenograft response with the clinical response of the donor patient (Steel *et al.*, 1983). However apart from the fact that the pharmacokinetics of anticancer drugs in tumour-bearing mice are poorly understood, there are also difficulties relating to the scheduling of treatment and the method of quantitating tumour response. Tumour growth delay can be influenced by such factors as the dosage and scheduling of drugs used, the site of the tumour and the immune response (Steel & Peckham, 1980; Warenius *et al.*, 1980). These problems are reflected in the results of therapeutic testing where at times a discordant therapeutic response between patient and xenografted tumour was seen. It was possible to compare response to chemotherapy of 7 xenografted tumours with that of the patient from which the xenografts were derived. In 4 instances there was a good correlation between responses observed while in 3 cases the results of treatment were somewhat different. The latter included; one patient who had no evidence of tumour following treatment with cisplatinum and cyclophosphamide yet the xenograft exhibited only minimal growth delay with cisplatinum and melphalan; one patient with a tumour clinically resistant to cisplatinum and chlorambucil, the growth of which in nude mice was temporarily inhibited during therapy but regrew rapidly after treatment; and one patient whose tumour was clinically resistant to cisplatinum and chlorambucil but where the xenograft responded completely to cisplatinum. There were some differences however, in the treatment of patients and xenografts as only single agents at the maximum tolerated dose were used in the xenograft system while patients usually received combination chemotherapy. Furthermore, the bulk of tumour present in patients differed from that in xenografts, a factor which may influence response to treatment.

Two of the three ovarian tumour lines propagated in tissue culture prior to being established as xenografts responded to therapy differently to that seen in the patient. It is conceivable that the genotypic and phenotypic changes which occur during cell culture could account for this difference. It has been previously claimed that xenografts may be useful in the selection of appropriate chemotherapy of individual patients (Giovanella *et al.*, 1983). However, the relatively low take rates, the long delay between establishing and subsequently testing xenografts, and the not uncommon paradoxical results seen with treatment would appear to preclude xenografted tumours having a predictive role in the choice of treatment for individual patients. In the therapeutic setting, the place of ovarian xenografts may be rather the primary screening of new drugs and testing novel experimental approaches to treatment such as hormonal therapy or the Mullerian Inhibitory Substance.

The major differences between xenografted and human ovarian tumours include the very low incidence of metastases in nude mice, and a tumour doubling time (mean = 14 days) that is far more rapid than already seen clinically (Steel & Peckham, 1980). The reasons for these differences are not known but it has been postulated that tumours that grow in mice are either selected for rapid growth or increase their growth rate in the new environment. Although the S phase and labelling indices are slightly higher in the xenografted tumour than in the original tumour, the differences are not large enough to explain the differences in doubling time, and a decreased cell loss factor may be important contributing factor (Rofstadt *et al.*, 1982). In addition, the xenograft model is a highly selective system which favours growth of tumours that in most instances are aggressive, aneuploid, poorly differentiated and disseminated at the time of diagnosis. These selection pressures make this model system unsuitable for investigating the biology of the whole spectrum of ovarian epithelial tumours. Only one diploid line from a patient with a Stage Ia ovarian cancer was established. While it is possible that this line may be a useful model for further study the fact that it grows as a xenograft may indicate that it is biologically different to the group of diploid ovarian tumours previously reported to have a relatively good prognosis. Further attempts to establish "biologically indolent" tumours are warranted as there are reports in other tumour types of benign and low grade tumours being successfully implanted (McManus *et al.*, 1978).

In conclusion, the findings of this study suggest that ovarian tumour xenografts, in general, reflect the biological characteristics of the tumours from which they are derived. Notwithstanding the number of limitations, tumour xenografts will be a useful model for exploring some of the biological properties of human ovarian cancer. Possible directions for future studies using xenografts include investigation into the degree of genetic instability, mutation rate, oncogene expression and acquisition of drug resistance in xenograft lines established from tumours that vary in their clinical behaviour.

The authors thank Judy Hood for typing this manuscript, Pat Gregory and Jenny Leary for expert technical assistance and the Gynaecologists at King George V Memorial Hospital and the Westmead Centre for providing tumour specimens. Stuart Davies gave invaluable advice on immunocytochemistry.

### References

ASHALL, F., BRAMWELL, M.E. & HARRIS, H. (1982). A new marker for human cancer cells. 1. The Ca antigen and the Cal antibody. *Lancet*, **ii**, 2.

BIGNER, S.H., MARK, J. & BIGNER, D.D. (1984). Karyotypic evolution of human glioblastoma multiforme (HGM) during establishment in culture. *Proc. Am. Assoc. Cancer Res.*, **36**, 142. (Abst).

DAVY, M., BRUSTAD, T & MOSSIGE, J. (1977). Irradiation of human ovarian tumours in nude mice. In: *Proceedings of Second International Workshop on Nude Mice*. Nomura *et al.* (eds), Stuttgart: G. Fisher Verlag, p. 491.

FRIEDLANDER, M.L., TAYLOR, I.W., RUSSELL, P. HEDLEY, D.W., MUSGROVE, E. & TATTERSALL, M.H.N. (1983). Ploidy as a prognostic factor in ovarian cancer. *Int. J. Gynecol. Pathol.*, **2**, 55.

FRIEDLANDER, M.L., HEDLEY, D.W., TAYLOR, I.W., RUSSELL, P. COATES, A.S. & TATTERSALL, M.H.N. (1984a). The influence of cellular DNA content on survival in advanced ovarian cancer. *Cancer Res.*, **44**, 397.

FRIEDLANDER, M.L., TAYLOR, I.W., RUSSELL, P. & TATTERSALL, M.H.N. (1984d). Cellular DNA Content – A stable feature in ovarian cancer. *Br. J. Cancer.*, **49**, 173.

FRIEDLANDER, M.L., RUSSELL, P., TAYLOR, I.W., HEDLEY, D.W. & TATTERSALL, M.H.N. (1984b). Flow cytometric analysis of cellular DNA content as an adjunct to the diagnosis of ovarian tumours of borderline malignancy. *Pathology*, **16**, 301.

FRIEDLANDER, M.L., HEDLEY, D.W. & TAYLOR, I.W. (1984c). The clinical and biological significance of aneuploidy in human tumours: A review. *J. Clin. Pathol.*, **37**, 961.

FUSENIG, N.E., BREITKREUTZ, DZARLIEVA, R.T., *et al.* (1982). Epidermal cell differentiation and malignant transformation in culture. *Cancer Forum*, **6**, 209.

GIOVANELLA, B.C., STEHLIN, J.S., SHEPARD, C.C. & WILLIAMS, L.J. (1983). Correlation between response to chemotherapy of human tumours in patients and nude mice. *Cancer*, **52**, 1146.

GOLDACRE, R.J. & SYLVEN, B. (1959). A rapid method for studying tumour blood supply using systemic dyes. *Nature*, **184**, 63.

HEYDERMAN, E., STEEL, K. & ORMEROD, M.G. (1979). A new antigen on the epithelial membrane: its immunoperoxidase localisation in normal and neoplastic tissues. *J. Clin. Pathol.*, **32**, 35.

HOUGHTON, J.A. & TAYLOR, D.M. (1978). Maintenance of biological and biochemical characteristics of human colorectal tumours during serial passage in immune deprived mice. *Br. J. Cancer*, **37**, 199.

KABAWAT, S.E., BAST, R.C., WELCH, W.R., KNAPP, R.C. & COLUIN, R.B. (1983). Immunopathologic characterization of monoclonal antibody that recognizes common surface antigens of human ovarian tumours of serous, endometroid and clear cell types. *Am. J. Clin. Pathol.*, **79**, 98.

KIM, W., TAKAHASHI, T., NISSELBAUN, J.S. & LEWIS, J.L. (1978). Heterotransplantation of human choriocarcinoma in nude mice. 1. Morphologic and biologic characteristics. *Gynecol. Oncol.*, **6**, 165.

KULLANDER, S., RAUSING, A. & TROPE, C. (1978). Human ovarian tumours heterotransplanted to nude mice. *Acta Obstet. Gynecol. Scand.*, **57**, 149.

LINDENBERGER, J. (1981). Aspects of xenografted tumours of the ear, nose and throat. Morphology, cell kinetics, growth behaviour and immunology. In: *Thymusaplastic Nude Mice and Rats in Clinical Oncology*. Bastert (ed.), p. 449. Stuttgart: Gustav Fischer Verlag.

McMANUS, M.J., DEMBROSKE, S.E., PIENKOWSKI, M.M. & 5 others (1978). Successful transplantation of human benign breast tumours into the athymic nude mouse and demonstration of enhanced DNA synthesis by human placental lactogen. *Cancer Res.*, **38**, 2343.

MILLER, A.B. HOOGSTRATEN, B., STAQUET, M. & WINKLER, A. (1981). Reporting results of cancer treatment. *Cancer*, **47**, 207.

POVLSEN, C.O. (1976). Heterotransplantation of human malignant melanomas to the mouse mutant nude. *Acta. Pathol. Microbiol. Scand* [*A*]., **84**, 9.

POVLSEN, C.O. & JACOBSEN, G.K. (1975). Chemotherapy of human malignant melanoma transplanted in the nude mouse. *Cancer Res.,* **35,** 2790.

POVLSEN, C., RYGAARD, J. & FOGH, J. (1982). Long term growth of human tumours in nude mice: evaluation of stability. In *The Nude Mouse in Experimental and Clinical Research,* Vol. 2, p. 79. Academic Press Inc.

POVLSEN, C.O., VISFELDT, J., RYGAARD, J. & JENSON, G. (1975). Growth patterns and chromosome constitutions of human malignant tumours after long term serial transplantation in nude mice. *Acta. Pathol. Microbiol. Scand.* [*A*]., **83,** 709.

RAGHAVAN, D., FRIEDLANDER, M.L. & RUSSELL, P. (1984). Tumour markers: applications in the management of cancer of the female genital tract. In: *Cancer Investigation and Management Series.* Whitehouse & Williams (eds.) (in press).

RAGHAVAN, D., GIBBS, J., NOGUEIRA, C. & 4 others (1980). The interpretation of marker protein assays. A critical appraisal in clinical studies and a xenograft model. *Br. J. Cancer,* **41** (Suppl. 4), 191.

RAGHAVAN, D., HEYDERMAN, E., GIBBS, J., NEVILLE, A. and PECKHAM, M. (1981). Functional and morphological aspects of human teratoma xenografts. In: *Thymusaplastic Nude Mice and Rats in Clinical Oncology.* Bastert *et al.* (eds.), p. 439, New York, Stuttgart: Gustav Fischer Verlag.

REEVES, B.R. & HOUGHTON, J.A. (1978). Serial cytogenetic studies of human colonic tumour xenografts. *Br. J. Cancer.,* **37,** 612.

ROFSTADT, E.K., FODSTAD, O. & LINDMO, T. (1982). Growth characteristics of human melanoma xenografts. *Cell Tissue Kinet.,* **15,** 545.

SELBY, P.J., HEYDERMAN, E., GIBBS, J. & PECKHAM, M.J. (1979). A human testicular teratoma serially transplanted in immune-deprived mice. *Br. J. Cancer,* **39,** 578.

SHIMOSATO, Y., KAMEYA, T. & HIROSHASHI, S. (1979). Growth, morphology and function of xenotransplanted human tumors. In *Pathology Annual Part 2.* Sommers & Rosen (eds.), p. 215, Crofts/New York: Appleton-Century.

STEEL, G.G., COURTENAY, V.D. & PECKHAM, M.J. (1983). The response to chemotherapy of a variety of human tumour xenografts. *Br. J. Cancer,* **47,** 1.

STEEL, G.G. & PECKHAM, M.J. (1980). Tumour xenografts: A critical appraisal. *Br. J. Cancer,* **41** (Suppl. 4), 133.

TATTERSALL, M.H.N. (1981). Pharmacology and selection of cytotoxic drugs. In: *Gynecologic Oncology Fundamental Principles and Clinical Practice.* Coppleson (ed.), Vol. 1, p. 121.

TAYLOR, I.W. (1980). A rapid single step staining technique for DNA analysis by flow microfluorimetry. *J. Histochem. Cytochem.,* **28,** 1021.

TAYLOR, I.W. & MILTHORPE, B.K. (1980). An evaluation of DNA fluorochromes, staining techniques and analysis for flow cytometry. I. Unperturbed Cell Populations. *J. Histochem. Cytochem.,* **28,** 1224.

TUEFEL, *et al.* (1981). Growth of human ovarian carcinomas in Thymusaplastic nu/nu mice. In: *Thymusaplastic Nude Mice and Rats.* Bastert *et al.* (eds.), p. 120. New York, Stuttgart: Gustav Fischer Verlag.

TILGEN, W., BOUKAMP, P., BREITKREUTZ, D., *et al.* (1983). Preservation of morphological, functional and karyotypic traits during long term culture and *in vivo* passage of two human skin squamous cell carcinomas. *Cancer Res.,* **43,** 5995.

VAN HAAFTEN-DAY, C., RUSSELL, P., RUGG, C., WILLS, E.J. & TATTERSALL, M.H.N. (1983). Flow cytometric and morphological studies of ovarian cell lines and xenografts. *Cancer Res.,* **43,** 3725.

WARENIUS, H.M., FREEDMAN, L.S. & BLEEHEN, N.M. (1980). The response of a human tumour xenograft to chemotherapy: Intrinsic variation between tumours and its significance in planning experiments. *Br. J. Cancer,* **41** (Suppl. 4), 128.